# Mesenchymal Stromal Cell Therapeutic Delivery: Translational Challenges to Clinical Application

**DOI:** 10.3389/fimmu.2019.01645

**Published:** 2019-07-31

**Authors:** Henry Caplan, Scott D. Olson, Akshita Kumar, Mitchell George, Karthik S. Prabhakara, Pamela Wenzel, Supinder Bedi, Naama E. Toledano-Furman, Fabio Triolo, Julian Kamhieh-Milz, Guido Moll, Charles S. Cox

**Affiliations:** ^1^Department of Pediatric Surgery, McGovern Medical School, University of Texas Health Science Center at Houston, Houston, TX, United States; ^2^Department of Transfusion Medicine, Charité Universitätsmedizin Berlin, Corporate Member of Freie Universität Berlin, Humboldt-Universität zu Berlin, and Berlin Institute of Health (BIH), Berlin, Germany; ^3^BIH Center for Regenerative Therapies (BCRT), Charité Universitätsmedizin Berlin, Corporate Member of Freie Universität Berlin, Humboldt-Universität zu Berlin, and Berlin Institute of Health (BIH), Berlin, Germany

**Keywords:** cellular therapy, mesenchymal stromal cell, clinical translation, safety, cell delivery, hemocompatibility, complement, coagulation

## Abstract

For several decades, multipotent mesenchymal stromal cells (MSCs) have been extensively studied for their therapeutic potential across a wide range of diseases. In the preclinical setting, MSCs demonstrate consistent ability to promote tissue healing, down-regulate excessive inflammation and improve outcomes in animal models. Several proposed mechanisms of action have been posited and demonstrated across an array of *in vitro* models. However, translation into clinical practice has proven considerably more difficult. A number of prominent well-funded late-phase clinical trials have failed, thus calling out for new efforts to optimize product delivery in the clinical setting. In this review, we discuss novel topics critical to the successful translation of MSCs from pre-clinical to clinical applications. In particular, we focus on the major routes of cell delivery, aspects related to hemocompatibility, and potential safety concerns associated with MSC therapy in the different settings.

## Introduction

The study of multipotent mesenchymal stromal cells (MSCs) and their usefulness for treating human injury and disease is almost 40 years old and has evolved through a number of phases roughly defined by the most commonly proposed mechanisms of action (MoA) at work. Interest in MSCs began as a study of bone marrow stromal cells in the 1970s by Friedenstein and contemporaries (1). This grew into an interest in their osteogenic differentiation potential in the late 1980s, and later broadened to trilineage differentiation (bone, fat, cartilage) (2). Cell fusion was briefly considered as a possible mechanism of repair (3–6). Aside from MSCs, a multitude of other stem cell populations with distinct properties have been isolated from adult rodent and human tissue, including multipotent adult progenitor cells (MAPCs) (7). Today, many studies focus on paracrine growth and immunomodulatory factors as key mediators of MSC's therapeutic effect, identified to protect injured tissue and to encourage endogenous repair mechanisms (8). In 2006, the International Society for Cellular Therapy (ISCT) published their first position statement on defining minimal criteria for MSCs (9), followed by several updates mainly focusing on the refinement of standards for therapeutic efficacy (10–12). Efforts to further refine cell pharmacology and drug delivery are ongoing (13). Thus, it becomes apparent that MSC research has undergone numerous advancements over time, in order to understand and benefit from the interesting properties of these cells. In this review, we will discuss key translational hurdles to clinical applications of MSCs. We will first outline popular cell delivery methods specific to their clinical application and then address newly identified efficacy and safety concerns regarding specific delivery methods (14).

## Routes of Therapeutic Cell Delivery

**Figure 1:**
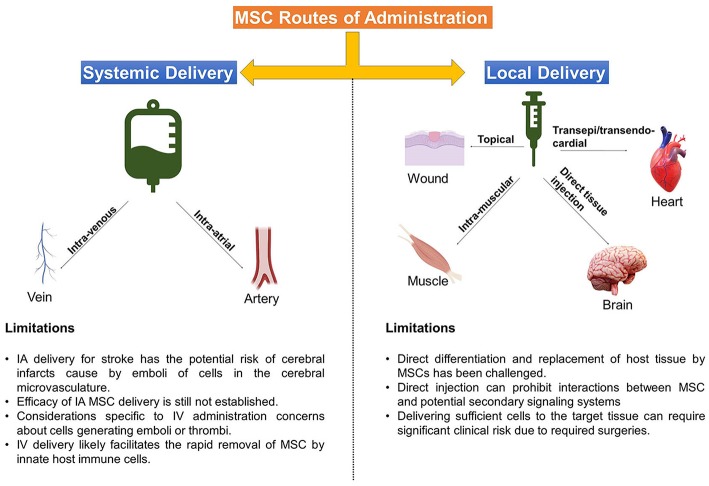
Delivery Routes Common for MSC therapies. Depicted above are the main methods that MSC are administered to target tissues, accompanied by some limitations of each approach.

A number of notable efforts have been made to compare the efficacy of different routes of MSC administration, which has become increasingly difficult with the large number of pre-clinical and clinical studies that are being published daily (14–18). Despite a number of direct comparisons in animal models and efforts to compare specific routes in a limited number of clinical trials, there is no consensus on the optimal method for MSC delivery (Figure 1), with specific limitations or advantages being associated with either method in clinical situations. Numerous methods for delivery of MSCs exist today and it stands to reason that different clinical indications and pathologies will require different delivery routes for optimal therapeutic efficacy (19, 20). Notably, a number of efforts are being made to develop MSC-derived exosomes or extracellular vesicles as a new “cell-free” way to recapitulate MSC activity with unique challenges and considerations (21).

Many investigators and industry driven studies rely on practical or logistical considerations (22). Nonetheless, new data-driven approaches for product optimization are currently being tested that integrate desired therapeutic MSC properties with representative simulated microenvironment interactions *in vitro* in an effort to determine optimal delivery and improve clinical outcomes (23–25). Selecting a suitable delivery route for future studies should also include a consideration of the desired MoA, and whether MSC culture techniques sufficiently highlight that mechanism, and if MSCs can be better primed by an alternative method (26).

Here, we will briefly discuss the rationale behind the most common delivery methods –topical application, intra-muscular (IM) or direct injection (DI) into tissues/organs, intra-venous (IV) infusion, and intra-arterial (IA) infusion, followed by notable considerations and translational challenges from preclinical to clinical application.

### Topical Application and Local Injection

Classically, localized topical application or injection of a cell therapy into a specific site or target tissue, e.g., intramuscular or penumbral area of an injury, has been shown to be very useful for precision delivery of MSCs and to increase the engraftment of therapeutic cells at a specific site of interest (14, 27). These strategies are often associated with a tissue replacement strategy or direct paracrine support as a MoA and can be particularly useful when combined with specifically tailored exogenous support systems and biomaterials to guide MSC-host interaction and encourage endogenous therapeutic actions (28, 29).

While the direct differentiation and replacement of host tissue by MSCs has been challenged as a result of a different activity, such as cell fusion or transfer of genetic material (6, 30–34), there are also some notable recent reports of MSCs directly contributing to tissue regeneration, such as in recent work in trachea and esophageal replacement (35, 36).

Topical application of MSCs is the least invasive method of delivery and has demonstrated great potential in the fields of burn medicine and wound care. Topically applied MSCs have improved outcomes, wound healing, and skin graft survival in burn wounds, diabetic-related wounds, and other chronic wounds (37, 38). Using a fibrin polymer spray system, Falanga et al. demonstrated that topically applied MSCs improved wound closure rates in a preclinical model, as well as in patients with chronic non-healing lower extremity wounds (39).

Intra-muscular (IM) delivery of MSCs, like topical application, presents a safe and simple method for cell delivery and, furthermore, leads to improved dwell time compared to other routes such as IV, intra-peritoneal (IP) and subcutaneous cell delivery (40). In the study by Braid et al. IM delivery of MSCs in a mouse model led to survival of human MSCs for up to 5 months after injection. In addition to extended dwell time, IM skeletal muscle fibers provide a highly vascular conduit for local and systemic release of trophic factors and support for MSC paracrine actions (27).

In critical limb ischemia (CLI), for example, MSCs may exert their restorative effects via promotion of angiogenesis and revascularization of ischemic tissue (41). A recent Cochrane analysis of autologous cells treatments, including bone marrow (BM)-MSCs, for CLI found no differences between IA and IM deliveries (42). Furthermore, Soria et al. found that IM delivery may be superior to IA delivery regarding the mitigation of adipose tissue (AT)-derived MSCs prothrombotic properties (43). Interestingly, work by Lataillade et al. has also shown promising effects of local IM-injections of MSCs in dosimetry-guided surgery treatment of radiations burns (44), while both, local IM and systemic IV delivery of MSCs and MSC-like cells has led to rescue from lethal radiation in animal models (45, 46).

In addition to topical and IM delivery, early investigative efforts often focused on the potential of MSCs to repair tissues by local engraftment and/or differentiation via direct injection (DI) into the target tissue or organ. Pre-clinical studies in neurological disease, such as stroke, attributed the beneficial effects of MSCs to their ability to engraft and differentiate into neurons and/or glia (47). However, the notion that MSCs can differentiate into functioning neuronal cells was subsequently challenged and appears unlikely (48–50).

Others contended that MSC engraftment facilitated endogenous neurorestorative mechanisms such as promotion of host neural and glial cell remodeling (51, 52). Regardless of the MoA, DI has potential advantage of bypassing the blood-brain barrier to increase delivery of cells into the central nervous system (CNS). For example, a recent Phase 1/2a clinical trial investigated intra-cerebral implantation of the SB623 MSC cell line in adults with chronic, non-hemorrhagic stroke via magnetic resonance imaging (MRI) stereotactic guidance into the peri-infarct area (53). The authors concluded that MSC implantation was safe, feasible and also improved neurologic outcomes at 12 months. However, important to note, is that the study was limited by patient selection (only 4.7% of screened patients were enrolled) and a lack of a control/placebo group.

Direct injection (DI) of MSCs has also been widely utilized for the treatment of cardiac disease—both acute myocardial infarction (AMI) and ischemic heart failure (IHF)—via open trans-epicardial and catheter-based trans-endocardial injection. There have been numerous preclinical and clinical trials in recent years, but here we will highlight only a few selected studies. Important to note, the notion that MSCs can differentiate into cardiomyocytes or promote cardiac stem cell proliferation and differentiation have largely been abandoned in the past decade. Furthermore, even the existence of resident adult cardiac stem cells has recently been challenged and appears unlikely (54, 55).

The PROMETHEUS trial investigated the use of intra-myocardial MSC injections into non-revascularized ischemic myocardium in patients undergoing coronary artery bypass grafting (CABG) for IHF and found improvements in regional myocardial and global left ventricular (LV) function (56). The authors rationalized the use of intra-myocardial injections based on the theory the MSCs exert their effects predominately at the injection site via release of anti-fibrotic matrix metaloproteases and stimulation of neovascularization.

Indeed, the authors found that the effect of MSC injection dropped off as a function of distance from the injection site. Of note, this study involved only 6 patients and no control group. However, a recently published, randomized trial of intra-myocardial injection of mesenchymal precursor cells (MPCs) in 159 patients with advanced heart failure undergoing left ventricular assist device (LVAD) placement found that MPC therapy did not demonstrate improvement in the primary outcome, weaning from LVAD support within 6 months (57). The authors also noted that one potential factor for the lack of efficacy may have been the use of trans-epicardial injections, which can lead to significant cell loss.

A systematic review and meta-analysis of MSC delivery methods in preclinical and clinical AMI found that trans-endocardial injections produced more favorable results in swine models in comparison to direct implantation (intra-myocardial) (58). Furthermore, the trans-endocardial approach allows for a minimally invasive, catheter-based direct implantation of cells into the myocardium and avoids the invasive thoracotomy, and thus additional risks for patient harm, required for trans-epicardial delivery.

There are several significant risks and considerations unique to DI delivery of MSC. Among them are reports of MSC differentiating into problematic tissue/ectopic tissue formation (59, 60), particularly heterotopic ossification into ectopic bone (61, 62). Additionally, localized DI may prohibit interactions between therapeutic MSCs and potential host secondary signaling systems in the lung, spleen, and peripheral blood, thus limiting their repertoire of therapeutic MoA. Consideration must also be given to the logistics and feasibility of various DI approaches, as delivering sufficiently high cell numbers to the selected target tissue can create significant clinical risk due to required surgeries, such as laminectomies to treat spinal cord injuries (63).

### Intra-arterial Infusion

There is substantial evidence that MSCs exert their effects largely via direct cell contact and local paracrine effects, as opposed to engraftment and differentiation within the target organ (64, 65). Additionally, upon systemic infusion, the interaction with endogenous inflammatory and tissue repair signals, such as immune cells and the innate immune cascade systems in the bloodstream likely influences MSCs responses, bio-distribution and homing to injured or diseased tissues (14, 66).

Intra-arterial (IA) delivery may prove the most efficacious method in one treatment indication, but may be potentially harmful in another. IA delivery of MSCs allows for infusion of cells within the local vascular system of the target organ without the physical risks of direct implantation and pitfalls of IV administration, especially the trapping of cells within the lung microvasculature, and may thus allow more cells to reach the intended target tissue (17, 65, 67).

Importantly, based on a survey of published results (68), the IA delivery of MSCs for stroke entails the potential risk of cerebral infarcts, caused by emboli of cells in the cerebral microvasculature. Factors such as vascular access, cell size, cell dosage and delivery speed must be considered, especially when delivering cells into coronary or cerebral arteries (69, 70).

In AMI several clinical trials have demonstrated safety and improvements in functional outcomes with the use of intracoronary infusion of MSCs and other BM cell populations (71–73). Here, IA delivery is likely a valid option, as it avoids invasive procedures (which are not part of the usual care in AMI) and ensures delivery of cells to the area of focal tissue injury and hypoxia.

As mentioned above, the SafeCell Heart study demonstrated significant improvements in LVEF with intracoronary MSC delivery (74). IA cell delivery has also been utilized in other pathologies including, but not limited to intra-carotid delivery in stroke, intra-renal delivery for renovascular disease, and intra-hepatic delivery for cirrhosis (75–77). However, the efficacy of IA MSC delivery in the above examples is still under investigation.

### Intra-venous Infusion

The most commonly used method to apply MSCs is via IV infusion, due to the relative ease and limited risk (14). This method results in a large number of MSCs accumulating in the lungs, but also distributing throughout the body and other organs, such as the spleen, throughout 24–48 h (67, 78–80). Similar to intra-arterial delivery of cell therapies, IV administration is most often associated with mechanisms involving secondary signaling effector cell systems and interactions with the host immune system (64, 80, 81).

Most of the attempts at developing a commercial cell therapy have used IV administration in order to facilitate use at multiple centers. Perhaps most widely known, the systemically IV-infused Remestemcel-L (Prochymal) has been developed by Osiris Therapeutics (Now Mesoblast), to primarily treat graft-vs.-host disease (GvHD) (82). Other notable efforts also include the use of Multistem, a multipotent adult progenitor cell that is somewhat similar to MSCs, to treat ischemic stroke (83) and our own use of cell therapies to treat traumatic brain injury (84). Common among these approaches is the use of cell therapies to modulate inflammation and activation of the immune system in order to decrease inflammation-related secondary injuries and restore homeostasis.

Among the considerations specific to IV administration are the same concerns about cells generating emboli or thrombi, however, with the advantage of the lung capturing potential vascular obstructions before they can disrupt other organ function. IV infusion certainly may result in limited numbers of cells reaching target tissues, a more transient persistence of cells, and a dilution of paracrine factors reaching target tissues. Finally, while it may be part of MSCs MoA in modulating immune responses (85), IV delivery likely facilitates the rapid removal of clinical MSCs by innate host immune cells (14, 86).

### Additional Routes of Administration

Several additional routes of MSC administration are notable for consideration for some specific applications. Intra-nasal (IN) administration of MSCs is promising route to treat neurologic pathologies that avoids the risks associated with direct injection of cells into the CNS. Preclinical data has demonstrated efficacy of IN MSCs across a spectrum of CNS disorders, including perinatal ischemic brain injury, subarachnoid hemorrhage, and neurodegenerative disorders such as Parkinson's disease (20, 87–90).

Intrathecal (IT) administration of MSCs, often through lumbar puncture, has shown benefit in preclinical and clinical studies across a wide array of neurologic disorders, including chronic neuropathic pain secondary to spinal cord injury (91–94), amyotrophic lateral sclerosis (ALS) (95–97), and epilepsy (98, 99).

Intravitreal administration of MSCs has demonstrated improvement in outcomes in several animal models of retinal injury and dysfunction (100–104); however, the safety of intravitreal injection of MSCs in humans is still an area of clinical concern (105).

Furthermore, the administration of MSCs during *ex vivo* perfusion of solid organs, e.g., in kidney transplantation or injury, avoids the trapping of cells in the lung and spleen and permits direct interaction of MSCs with the target tissue (106). Gregorini et al. demonstrated that delivering MSCs during hypothermic machine perfusion improved outcomes in a rat model of ischemic kidney injury (107). Recently, Sierra Parraga et al. demonstrated that machine perfusion of MSCs supports their function and survival, although more work will be required to determine the optimal conditions for perfusion (108). Notably, these strategies may reduce the need for immunosuppression to prevent organ rejection (109).

## Translational Hurdles With Systemic and Local Delivery

**Figure 2:**
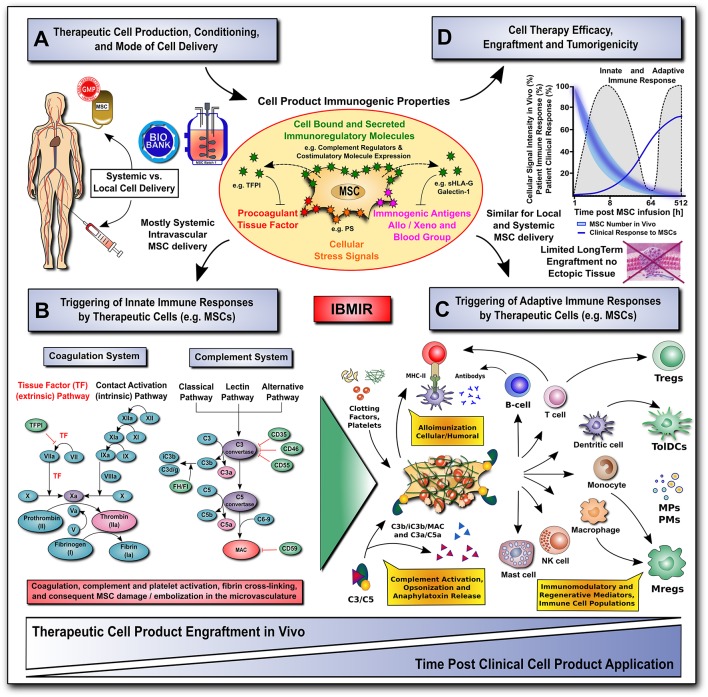
Translational challenges with systemic and local cell delivery. **(A-D)** Therapeutic cell production / conditioning (e.g., 2D vs. 3D culture and cytokine priming) and the mode of cell delivery (e.g., systemic intravascular infusion vs. local injection) have a major impact on the cell product's immunogenic properties (shown in **A**), and consequent rapid triggering of innate and adaptive immune responses (shown in **B,C**), thus affecting its therapeutic efficacy, engraftment and tumorigenicity (shown in **D**). The MSC product's immunogenic properties are affected by numerous cell-bound and secreted immunoregulatory mediators (e.g., complement regulators, coagulation regulator TFPI, or regulators of the adaptive immune response, such as co-stimulatory molecule expression, sHLA-G and galectin-1). The cells can also exhibit a number of immunogenic features, such as procoagulant TF-expression, cellular stress signals (e.g., PS), and immunogenic antigens (e.g., allo, xeno, and blood groups). **(B)** The innate coagulation and complement cascade systems are two of the major effector arms of the instant blood-mediated inflammatory reaction (IBMIR) that can recognize blood-incompatible therapeutic cell features and thus trigger the detrimental thromboinflammation compromising cellular therapeutics. The innate immune cascade systems employ multiple sophisticated molecular sensors (e.g., FVII and FXII, or C1q and MBL, respectively), to recognize aberrant cell surface molecular features on infused therapeutic cells (e.g., TF and PS, respectively), which can trigger innate immune cascade activation and amplification by effector cells (e.g., platelets, PMNs, monocytes/macrophages, and T/B cells), potentially leading to adverse reactions (e.g., cell lysis, inflammation, sequestration and rejection). **(C)** Innate and adaptive effector cell modulation: triggering of IBMIR and therapeutic cell injury/disintegration promotes the release of various bioactive molecules, in itself and from dying MSCs, upon crosstalk with the responsive host immune system, such as activated clotting factors (e.g., thrombin), anaphylatoxins (C3a and C5a), opsonins (iC3b, and C3d/g), and MSC-derived constituents (e.g., microparticles, cytokines and growth factors) in a highly conditional manner, thus greatly amplifying the initial signal, leading to modulation of multiple effector cell types. This can result in alloimunization and consecutive cellular and humoral responses (e.g., T-cells and B-cells alloantibodies), but also in the induction and release of multiple immunoregulatory and regenerative cell types and mediators (e.g., Tregs, Mregs, TolDCs, MPs, and PMs). **(D)** A large fraction of the infused therapeutic cells is lost within the first hours to days of infusion due to the triggering of instant innate immune responses, which can be furthermore aggravated by triggering of adaptive immune responses in case of allogeneic cell products. Studies on MSC persistence *in vivo* have shown prolonged survival, dwell-time, and engraftment by alternative routes of delivery (e.g., local injection in conjunction with biomaterials), although long-term engraftment is very limited and ectopic tissue formation rarely reported. Currently, patient clinical responses are still sub-optimal for many MSC therapeutics leaving room for improvement in long-term survival. AT, antithrombin; FI-FXII / FIa-FXIIa; native and activated coagulation factors I-XII; TF / TFPI, tissue factor and tissue factor pathway inhibitor; C3/5-9, complement component 3 and 5 to 9; C3a/C5a, activation fragment a of complement component 3 and 5; C3b/iC3b/C3d(g), complement component 3 sequential degradation fragments b, inactivated fragment b and d(g); complement regulatory molecules: CD35, complement receptor 1, CD46/MCP, membrane cofactor protein; CD55/DAF, decay accelerating factor; CD59, protectin; FI and FH, complement factor I and H; PS, phosphatidyl-serine; MAC, membrane attack complex; and MHC-II, major histocompatibility complex class-II; sHLA-G, soluble human leukocyte antigen G; MPs and PMs, MSC and immune cell-derived micro-particles and paracrine mediators; Tregs, Mregs and TolDCs, immunogerulatory T-cells, myeloid cells, and tolerogenic dendritic cells.

Any therapeutic modality, whether a new pharmacologic agent or surgical procedure, must not only be efficacious, but also safe for the patient. While the vast majority of preclinical and clinical studies have found MSCs to be safe and well-tolerated (110), the rise of unregulated and unproven stem cell interventions have resulted in several reported clinical adverse events (14, 60). Here we will highlight mechanisms by which MSCs may cause unwanted or adverse reactions, including interactions with the host's innate and adaptive immune system, as well as their tumorigenic potential (Figure 2).

### Triggering of Innate Immune Responses by MSCs

Systemically infused MSCs activate the host innate immune cascade systems, such as complement and coagulation, termed the instant blood-mediated inflammatory reaction (IBMIR) (14, 66, 111). IBMIR was first used to describe the procoagulant activity of pancreatic islet cells and hepatocytes in relation to their expression of procoagulant tissue factor (TF/CD142) (112, 113).

MSCs demonstrate similar effects when they contact human blood with variable amounts of procoagulant activity when isolated from different issues (14). They normally reside in the perivascular space around blood vessels, are predominantly excluded from direct contact with blood, and express variable amounts of procoagulant TF, first described on placenta-derived decidual stromal cells (DSCs) (114).

The effects of *ex vivo* expanded MSCs administered IV into patients include a significant increase in complement C3 activation fragment a (C3a) and the coagulation activation marker thrombin-antithrombin complex (TAT), which was accompanied by a decrease in platelet counts and a significant increase in fibrinolysis marker D-dimer (111, 115). Importantly, MSC's TF expression was shown to increase as cell passage increases, and the procoagulant effect of MSCs increases as their TF expression increases (14).

Furthermore, in a study investigating human BM- and AT-derived MSCs, TF expression and procoagulant activity were measured using flow cytometry and calibrated automated thrombogram and thromboelastography, respectively, demonstrating large donor and tissue variability (116). A causal relationship between MSC-associated TF with clot formation has also been demonstrated in human blood using flow cytometry to measure the density of TF on different types of MSCs (24), finding that MSC induce a dose-dependent change in clotting time and thrombin production based on TF expression.

Several authors demonstrated a reduction in procoagulant activity in MSCs when the cells were diluted or treated with TF pathway blocking reagents (24, 111, 117). Multiple authors also found that heparin can nullify accelerated clotting time due to MSC associated TF *in vitro* (115, 117–119). These data highlight the importance of monitoring MSC's procoagulant activity and provide a possible clinical solution. Thus, MSC-associated TF must be considered as a safety release criterion prior to administration in patients (14).

While a substantial number of *in vitro* investigations have demonstrated that MSCs exert a procoagulant effect after blood contact, there are limited reports of MSC-associated thrombotic events in humans in the literature, specifically in established clinical trials (60). One of the first reports involved the use of human placenta-derived MSC-like cells in a Phase 1b/2a study in Crohn's disease. The authors reported that two patients suffered from venous thrombosis after infusion (120), and posit TF expression on clinical-grade therapeutic MSCs as a possible cause (111).

Furthermore, subsequent *in vitro* studies comparing placenta-derived DSCs to BM-MSCs found a 15-fold higher expression of TF in the DSCs (115), which may help to explain the results from the above study. The need for increased caution with perinatal tissue (PT)-derived MSC products is further substantiated by a recent report of thromboembolism in two patients treated with umbilical cord MSC products (121).

Another key report of thrombotic events was in a trial that evaluated the use of autologous AT-derived MSCs to treat patients with critical limb ischemia (CLI) (122). The investigators found that two of the patients, both of whom also had diabetes, developed distal microthrombi after infusion. Interestingly, the investigators found no cases of thrombotic events when using autologous AT-MSCs in non-diabetic patients or autologous BM mononuclear cells (BM-MNCs) from diabetic patients.

It was subsequently demonstrated that AT-MSCs from diabetic patients release higher levels of plasminogen activator inhibitor type 1, reduced levels of tissue plasminogen activator, and lower d-dimer formation in comparison to non-diabetic AT-MSCs, all of which might lead to blunted fibrinolytic activity. Furthermore, these diabetic-derived AT-MSCs upregulated TF expression and displayed altered platelet-derived growth factor (PDGF) signaling, which was abrogated using PDGF-BB treatment (123).

There are other case reports of MSC-associated thrombotic events. A man who presented with chest pain was found to have small bilateral pulmonary emboli 1 month after receiving the last of multiple systemic infusions of AT–derived MSC for herniated cervical discs (124). His parents were reported to also have had MSC infusion for osteoarthritis, and both were found to have small pulmonary emboli, although neither was symptomatic. No evidence of hypercoagulable disease was found in any family member. There was no clear mention of the exact source of the clinical MSCs infused, or if these patients received MSCs in conjunction with a clinical trial. There is an additional media report of a 73 year old man who died from a pulmonary embolism after receiving an infusion of AT-derived stromal cells in Japan (125). It appears that this patient was not enrolled in any published clinical trial, and the company involved has come under intense scrutiny. In both of the latter two cases, there are few details regarding the source, manufacturing process and testing of the cells, as well as details surrounding the treating parties.

While the evidence for MSC-associated thrombotic events is nominal, there is a need to ensure the safety of MSC therapy, including their procoagulant effects (14). Furthermore, many patients who may benefit from MSC therapy—those with inflammatory mediated disorders, diabetes, cancer, cardiac dysfunction, or trauma-related injuries—are likely to have an acquired hypercoagulable state or are at high risk of a thrombotic event secondary to their primary disease process. Therefore, we must continue to monitor the pro-thrombotic effects of MSCs as part of release criteria and in clinical trials.

The complement system, another major part of the innate immune cascade systems, is one of the first lines of defense against foreign pathogens. It is not surprising that systemically infused MSCs activate and interact with the complement system (126). In fact, the complement system appears to play a critical role in initiating the immunomodulatory reaction between infused therapeutic MSCs and host cells (127). What role the complement system plays in MSC's MoA and how it affects their efficacy is currently under investigation.

Early studies by Tu et al. revealed that MSCs constitutively express factor H, a complement inhibitor (128); however, even when augmented by the presence of pro-inflammatory cytokines TNF-α and IFN-γ, the overall production of factor H by MSCs proved relatively insignificant in comparison to overall systemic levels. Subsequently, the same group and others demonstrated that although MSCs express cell-surface complement regulators, MSCs activate the complement system upon contact with human sera *in vitro*, leading to cytotoxicity in a dose-dependent manner (126, 129, 130).

Blockade of these complement inhibitors led to increased cytotoxicity, while both upregulation of CD55 (a cell-surface complement inhibitor) on MSCs via transfection with recombinant adenovirus, and addition of anti-C5 immunoglobulin significantly reduced MSCs cytotoxicity after contact with serum. Li et al. also determined allogeneic MSCs caused increased complement activation, with associated increased cytotoxicity, in comparison to autologous MSCs (126).

In order to prevent complement-mediated cytotoxicity against therapeutic cell products, investigators have engineered complement-resistant MSCs. Heparin is known to inhibit complement activity, and systemic administration of heparin reduced MSC cell damage after infusion (131). In order to prevent unwanted anti-coagulation effects of systemic heparin administration, the authors then demonstrated that incubation of MSCs with activated heparin led to binding of heparin to the surface of MSCs and furthermore, these “heparin-painted” MSCs showed less surface deposition of complement C3 activation fragment b (C3b/iC3b) and suffered less cell damage after contact with serum.

The proposed mechanism of heparin-mediated protection involves recruitment of factor H binding to the MSC cell-surface. Subsequent “painting” of factor H onto MSCs via pre-incubation led to decreased complement deposition onto MSCs surface, reduced cell damage, and increased cell survival *in vitro* and *in vivo* (132). In addition, MSCs with factor H incubation attenuated C5a release, which significantly reduced complement-mediated activation of neutrophils and led to improvement in MSC function and reduced cell damage.

Investigators have also engineered human MSCs via transduction of a retrovirus encoding genes from human cytomegalovirus (HCMV), which downregulated HLA1 expression on MSCs without increased vulnerability to NK killing (133). Furthermore, HCMV is known to incorporate the host-encoded complement inhibitor proteins and upregulate host-encoded CD55 and CD46 in order to evade the innate immune system. Soland et al demonstrated that induction of MSCs with the specific HCMV US2 protein led to upregulated expression of complement inhibitors CD46, CD55, and CD59, and a reduction in complement mediated MSC lysis (129).

While further investigation into engineered MSCs as described above is certainly necessary, the ability to evade or attenuate complement-mediated cell damage and lysis may prove crucial to the efficacy of future MSC therapeutics (86). Further investigation has demonstrated a much more complex relation between MSCs and the complement system, with discrepancies between pre-clinical and clinical results. In a clinical trial of patients receiving MSC infusions for treatment-resistant GvHD, the investigators examined the relationship between complement activation, immunosuppressive potential of the MSCs *in vitro* and the clinical effectiveness of MSC therapy *in vivo* (127). MSCs activation of the complement system was found to mediate effector cell activation and modulate their immunomodulatory activity in a multifactorial manner.

This finding was mechanistically substantiated by using *in vitro* inhibition of complement function, which resulted in decreased CD11b upregulation on effector cells. Furthermore, MSC's ability to activate the complement system was found to correlate with its immunosuppressive potential *in vitro*: MSCs with increased complement activating properties demonstrated increased suppression of peripheral blood monocyte (PBMC) proliferation in mixed lymphocyte reactions (MLRs) and increased ability to trigger CD11b+ effector cells in whole blood. Surprisingly, the authors found an inverse correlation between the immunosuppressive potential of the MSCs *in vitro* and their clinical effectiveness in human patients, with average suppressing cells yielding the most beneficial therapeutic effects *in vivo*.

Importantly, it appears that substantial differences exist with respect to complement activation and potential efficacy of fresh vs. freeze-thawed MSCs upon systemic infusion (86). Freeze thawed MSCs were found to demonstrate increased activation of the IBMIR and susceptibility to complement-mediate cell lysis (130). Similar changes in the immunomodulatory capacity of MSC due to cryopreservation have also been reported elsewhere: such as the alteration of MSC-mediated attenuation of T cell activation, inflammatory cytokine concentrations, and an increased susceptibility of MSC to lysis by mixed immune cells (134, 135).

In the clinical sample evaluated (130), the majority of GvHD patients were treated with freeze-thawed MSCs with a small number of fresh culture-derived MSCs being available for comparison; while the study lacked sufficient power and thus should be considered with caution, as also emphasized in the discussion, the authors noted an interesting trend of improved clinical outcomes with fresh MSCs, especially those delivered at early passage, in comparison to freeze-thawed MSCs (130, 136).

Overall, it is now evident that MSCs activate the complement system upon contact with blood, which appears to be positively correlated with their immunosuppressive ability *in vitro*. Furthermore, freeze-thawed MSCs, in comparison to fresh MSCs, demonstrated an increased triggering of the IBMIR and associated complement-mediated lysis *in vitro*, leading to a significant reduction in viable cells (130, 137). What remains unanswered is: (1) how and to what extent complement activation influences the clinical efficacy of systemic MSC therapy, and (2) whether fresh MSCs are subject to decreased complement-mediated lysis and, as a result, are more effective clinically than freeze-thawed MSCs *in vivo*.

### Triggering of Adaptive Immune Responses: Autologous vs. Allogeneic MSCs

A comprehensive review of the immunobiology of MSCs and the many ways that MSCs interact with the local and systemic immune system in both normal and activated systems is beyond the scope of this review, and has previously been covered in several notable efforts to consolidate the literature (138–141). It would not be an understatement to say that MSCs have extensive possible interactions with every major component of the immune system through a combination of paracrine activity, extracellular matrix remodeling, direct contact-based signaling, and more recently, through the use of extracellular vesicles.

These wide-ranging putative molecular mechanisms have made it incredibly difficult for the field to come to a meaningful consensus regarding the effects of self vs. donor antigens, further complicated by the additional xenogenic antigens introduced during common cell culture techniques (such as expansion in FBS) (137, 142, 143). Until recently, MSCs were widely reported to be immune privileged, enabling their use as an allogeneic therapy without concurrent immunosuppression.

As the field increasingly focused on the immunobiology of MSCs, there was a correlating rise in the number of studies that found that MSCs were not exempt from immune recognition. As summarized in a number of more comprehensive literature reviews (140, 144–146), allogeneic MSCs with poorly matched HLA can and do generate both innate and humoral responses from the immune system, albeit responses that appear to be dependent upon the conditional expression and balance of both immune-activating antigens (such as MHCs) and immune-modifying cytokines, molecules and metabolites, like tumor necrosis factor-inducible gene 6 (TSG-6), galectin-1, prostaglandin E2 (PGE_2_), and indoleamine 2,3-dioxygenase (IDO).

The eventual immune recognition of allogeneic and HLA-mismatched MSCs has become increasingly implicated as a barrier to clinical efficacy (140). On the one hand, the huge number of pre-clinical studies in both xenogeneic and allogeneic systems and clinical studies using allogeneic cells without regard to conventional graft-vs.-host compatibility considerations indicate that MSC efficacy is often independent of the eventual immune “rejection” of donor MSCs, either by virtue of MSC activity occurring prior to immune recognition or perhaps even due to a MoA that includes the host immune system.

In a recent report it was found that MSCs can modulate the immune system by being engulfed by antigen presenting cells, and that the subsequent display of MSC antigens by antigen-presenting cells (APCs) results in a chain of anti-inflammatory activity and downstream therapeutic outcomes (85). On the other hand, the recognition and removal of MSCs by the host immune system may also limit the duration and possible efficacy of a number of MSC MoAs.

Among a number of strategies to evade immune recognition, there is a large amount of interest in the use of biomaterials and engineering techniques to shield MSCs from immune activity to prolong paracrine activity (29, 147, 148), efforts to further decrease the immunogenicity of cells (149–151), sophisticated banking strategies to allow for autologous or HLA-matched cells to treat acute injuries in a timely fashion (152, 153), and conventional pharmaceutical immunosuppression given temporarily, all to provide a larger window for MSC activity *in vivo* (154, 155).

It is our opinion and that of others (86, 136, 140, 156), that reducing the activity of the host immune system is likely to also reduce the therapeutic efficacy of MSCs, as we feel that many of the pleiotropic effects of MSCs require the participation of the host immune system.

### Cell Engraftment and Tumorigenicity

The potential for malignant transformation of MSCs is of obvious concern. Because MSC therapy involves *ex-vivo* production and expansion of cell lines, and even allogeneic MSCs have the capacity to escape elements of immune recognition, it is crucial to ensure that transplanted or infused cells do not contain transformed, potentially tumorigenic cells (157).

Concerns for the tumorigenic potential initially came from studies of mouse-derived MSCs transplanted into a mouse model (158). The murine-derived BM-MSCs used in the study were reported to spontaneously transform into malignant fibrosarcomas in multiple organs *in vivo* after systemic infusion into immunocompromised mice. Of note, this same study also evaluated human MSCs and found no evidence of malignant potential *in vitro*.

Another study demonstrated that the injection of mouse MSCs with spontaneous p53 mutations led to development of fibrosarcomas at the site of injection in immunocompetent mice (159). Yet, there was no evidence that the transfer of MSCs without such mutations led to tumor formation. Transformed MSCs have also been identified for other non-human species. The authenticity of such findings is difficult to confirm in non-human cells, as many of such studies lack true verification by modern stringent methods, such as short tandem repeat (STR) profiling (160).

Additional reports of spontaneous malignant transformation of human MSCs further intensified concerns for the tumorigenic potential of MSCs. Two separate studies identified spontaneous malignant transformations of human MSCs in culture, and injection of these transformed cells led the development of tumors in mice (161, 162). However, the findings from both of these studies have since been retracted, as the MSC cultures in both instances were found to have been contaminated with established malignant human cell lines (163).

While two more recent studies have demonstrated, and confirmed using STR analysis, the development of malignant transformation of MSCs in both cynomolgus macaques and human cell lines (164–166), there are, in contrast, far more studies that have demonstrated that *ex vivo* expanded human MSCs are rather resistant to malignant transformation, even after long-term culture, development of chromosomal aberrations, and application of physical and chemical stress (167–169), and that they undergo senescence rather than becoming tumorigenic.

Further reports concerning the genetic instability of MSCs appear to be grossly overstated and even misleading (170, 171). In addition, studies of Good Manufacturing Practices (GMP) grade MSCs have also demonstrated a lack of malignant potential *in vitro* and *in vivo* (172, 173). While the possibility of rare tumorigenic transformations in MSCs cannot be ignored, careful monitoring of cell cultures, minimization of *in vitro* expansion length and evaluation for cytogenic aberrations when concerns rise should be considered (174).

Human MSCs have been used clinically for more than two decades, the majority from BM, but also increasingly also from other sources. To date, there are no clinical studies that have attributed MSC therapy to the development of tumors or malignancy. A meta-analysis of 36 studies, including 8 randomized control trials (RCTs), involving 1,012 patients found no evidence of association between MSCs and tumor formation (110). A 2013 report from the ISCT noted that although the risk of tumorigenicity of MSCs had yet to be confirmed or denied, no tumors have been diagnosed in patients that would originate from administered MSCs (175). While it appears that MSC therapy is safe and well-tolerated in human subjects, the risk of tumorigenicity must continue to be studied both in clinical trials with long-term follow up and during the culture/expansion process prior to any therapeutic infusion.

## Comparative Studies for Optimal Delivery

Two important considerations exist in determining optimal cell delivery. The first consideration is patient safety. The second consideration is the efficacy of a therapy. If DI, IA, or IV demonstrate similar clinical effectiveness, then the least invasive method of cell delivery is preferred. But do MSCs work differently when delivered DI v. IA v. IV? Here we have selected a few examples of preclinical and clinical studies that have compared cell delivery methods head to head in order to demonstrate potential advantages/disadvantages of one method over another.

In heart disease, DI into myocardium may not provide improved MSC engraftment rates or outcomes over IA infusion. In a model of porcine model of ischemic cardiomyopathy, animals received either surgical implantation, trans-endocardial injection, or intracoronary infusion of autologous MSCs and were euthanized 4 h after infusion (176). DI of MSCs via surgical implantation or trans-endocardial injection led to only 16 and 11% retention of MSCs within the myocardium, respectively. The majority of the cells, around 45%, accumulated in non-target organs for all three delivery methods. Intracoronary infusion led to similar rates of intra-myocardial MSC retention as DI (both 11%). IA delivery necessitates the need for patent vasculature and, therefore, may not provide benefit in pathologies such as AMI or ischemic stroke secondary to occluded internal carotid or intra-cerebral arteries.

There have also been investigations of IA vs. IV cell delivery, especially in stroke, as endovascular treatments have become increasingly utilized (70). Byun et al. demonstrated that IA delivery leads to improved cerebral engraftment and outcomes over IV delivery in a rat model of cerebral infarction (177). A meta-analysis of preclinical studies of MSCs in ischemic stroke models found that although DI provided the greatest benefit, all 3 methods of delivery—DI, IA, and IV— consistently demonstrated significant improvement in outcomes (178).

Direct comparison of delivery methods is often lacking in clinical trials due to logistical concerns. Furthermore, pooled meta-analysis and systematic reviews often combine different cell types, related pathologies and delivery methods. Therefore, the following small selection of studies is limited and should not be considered a representative sample.

In stroke, a pooled analysis of clinical trials using multiple cells types, largely BM-MNCs and MSCs, Jeong et al. determined that IA provided increased benefit over DI or IV (179). Furthermore, the SafeCell Heart study, a systematic review and meta-analysis of cell therapy in heart disease, found that IA and intra-myocardial (catheter-directed trans-endocardial) infusions provided significant improvements in LVEF, which were not seen with trans-epicardial or IV cell delivery (74).

In another meta-analysis of preclinical and clinical studies of MSCs in cardiac disease, Kanelidis et al. found that cell delivery method did have an effect on outcomes in AMI, and that trans-endocardial and IV delivery improved outcomes in both swine models and clinical trials, while IA infusion with subsequent intracoronary delivery did not demonstrate significant benefit (58).

Clearly, the currently available data, based on few preclinical studies and limited clinical trials, which are often contradictory, are not sufficient to make any major conclusions as to whether one delivery method is superior to another. However, clinical trials directly comparing cell delivery methods will likely not happen until MSC therapy, via any delivery method, is proven efficacious for a particular pathology.

## Conclusion

During the past decades, MSC therapeutics have undergone a continuous transition from proof-of-concept to clinically approved therapies. In order to improve our ability to utilize MSCs therapies, great efforts are ongoing to refine potency assessment, cell pharmacology and drug delivery. Compared to advancements in cell sorting, manufacturing and biobanking, the importance of cell delivery methods and the *in vivo* effects of MSCs on the human immune and hematologic systems are still largely underappreciated today. Thus, we here discussed key aspects related to the effective and safe delivery of MSCs, in the context of recent clinical studies with focus on different methods of MSC administration. As the growth of MSC-based therapeutics accelerates in private, public, and fringe applications, it is vitally important to remember historical safety concerns, recognize modern clinical risks, and use methodology and delivery consistent with the intended MoA, in order to yield the most effective and safest economically viable therapeutic approaches. We encourage our colleagues to careful consider their assumptions and commonly used practices to ensure that their long-held views about MSC biology are supported by modern studies.

## Author Contributions

HC, SO, AK, MG, and PW wrote the manuscript. KP, JK-M, and GM worked on illustrations. HC, SO, AK, SB, NT-F, FT, GM, and CC edited the manuscript.

### Conflict of Interest Statement

SO has received research support from Athersys, CBR Systems, Hope Bio and Biostage. CC has received research support from Athersys, Cellvation, CBR Systems, Hope Bio, and Biostage, and is on the Scientific Advisory Board of Cellvation, Biostage, and CBR. The remaining authors declare that the research was conducted in the absence of any commercial or financial relationships that could be construed as a potential conflict of interest.
